# SIMPLIFIED CALCULATION FOR CORRECTIVE OSTEOTOMIES OF LONG BONES

**DOI:** 10.1590/1413-785220162405160466

**Published:** 2016

**Authors:** EPITÁCIO LEITE ROLIM, MARCELO RAUL CAVALCANTI TORRES, MAURISTON RENAN MARTINS SILVA, FILIPE RAMOS LIMA, JOSÉ LAMARTINE DE ANDRADE AGUIAR

**Affiliations:** 1. Universidade Federal de Pernambuco (UFPE), Department of Surgery, Recife, PE, Brazil.; 2. Hospital Getúlio Vargas (HGV), Department of Orthopedics and Traumatology, Pernambuco, PE, Brazil.

**Keywords:** Osteotomy/methods. Bone and bones/abnormalities. Surgical procedures, operative.

## Abstract

**Objectives:**

To present a simplified calculation for the measurement of osteotomy wedges used for the correction of angular uniplanar deformities of long bones and to compare the simplified calculation proposed (circumferential calculation) with the classical trigonometric calculations, as well as with the exact calculation performed by computer software AutoCADtm.

**Methods:**

The software AutoCADtm was used to calculate the bone wedges, for mathematical comparison of the three main groups, each one of them containing 18 hypothetical bone deformities which angles ranging from 5 to 90 degrees, with 5 degrees intervals between them.

**Results:**

In the analysis of 18 deformities, the hypothetical angular bone, the average lengths of the corrective wedges obtained by the trigonometric, circumferential and the exact metods were, respectively, 32.21 ± 16.81 mm, 33.16 ± 18.63 mm and 35.22 ± 23.52 mm. There was no statistically significant difference between the three calculation methods (p>0.05).

**Conclusion:**

The circumferential calculation proposed in this study is useful for being accurate and simple, not requiring any trigonometric knowledge. Level of Evidence II, Experimental Study.

## INTRODUCTION

Osteotomies have been used for decades as a surgical strategy for correction of deformities in long bones.^1^ ^-^ ^3^ Several studies have been performed throughout history, describing the use of osteotomies to restore the mechanical and anatomical axes of the long bones, to treat articular degenerative cases and to correct congenital deformities or vicious consolidations that compromise the function or aesthetics of pacients.^2^ ^-^ ^6^

In 1878, Macewen^1^ described a technique that became known as cuneiform osteotomy, using it at that time to correct genu valgus deformities.

Numerous techniques have been introduced since, including the following types of osteotomies: linear, circular locking, pivotal, z-shaped or telescope, all with advantages and disadvantages regarding fixation easiness, damage to soft tissues and capability of normal axis correction.^3^ ^,^ ^6^ ^,^ ^7^

Panoramic radiographs of the upper and lower limbs are essential for surgical planning regarding the correction of bone deformities, allowing predicting the interventions and calculations of the central point of the deformity and its correction axis, named, respectively, Center of Rotation of Angulation (CORA) and Angulation Correction Axis (ACA).^8^ ^-^ ^10^

For cuneiform osteotomies of long bones, the calculation of the wedge dimensions, whether a subtraction- or addition-type, have been classically performed using trigonometric methods, which ensures great precision, considering that cylinder wedges are geometrically compatible with triangles in the uniplanar analysis.^11^ ^-^ ^13^ Other forms of programming involve the direct method, in which the wedge dimensions are measured on the radiographic film, which also lead to good results, despite being more subject to deviations from the radiographic magnification, besides possible measurement errors that can affect the intervention's outcome.^14^

The use of the trigonometric method presents excellent accuracy, but involves the use of trigonometric tables on sine, cosine or tangent of the different angles needed to correct the deviations, which is not always well understood by orthopedic surgeons.^8^ ^-^ ^16^ Moreover, the use of subtraction wedges requires the definition of its base through the convex side of the deformity, and those are usually shaped as an arc of a circle, rather than a straight line that would represent the basis of a triangle.^11^ ^-^ ^16^

The direct measurement methods rely on the accuracy of measuring devices, such as calipers, rulers or measurement tape, and their correlation with the quality of radiographic films, besides having to be corrected for the magnification, which requires ratio calculations and radiopaque reference of known measures.^14^ ^,^ ^16^

The presentation of a simplified calculation for defining the base of the osteotomy wedge, which eliminates the use of trigonometric tables and involves only one constant and easily detectable variables (diameter of the bone and correction angle), besides using a magnification correction method with a radiopaque reference to standardized measures, may facilitate the surgical planning of corrective osteotomies of long bones.

The objectives of this study were to present a simplified calculation for measuring the osteotomy wedges used for correction uniplanar angular deformities in long bones and to compare the proposed simplified calculation (circumferential calculation) with the classic trigonometric calculations and the exact calculation through a computer software to define the dimensions of corrective wedges for the correction of angular deformities in long bones.

## MATERIALS AND METHODS

This was an experimental and descriptive study conducted at *Hospital Getúlio Vargas* and *Universidade Federal de Pernambuco* from January 2014 to August 2015. There were no conflicts of interest in this study, and the study type did not require approval by the Research Ethics Committee.

### Sample selection 

For the calculation and mathematical comparison of the diameter of the bone wedges, three main groups were created, modeled and simulated in AutoCAD^tm^, each of them containing 18 three-dimensional bone model sample of any long bone, without deformities (CORA zero in the mechanical axis), greater diaphyseal diameter of 40mm on the coronal axis. In the same software, a varus deformity at the diaphyseal level whose angles ranged 5-90° with 5° intervals between them was created, dividing, thus, the bone model in two segments of similar lengths was produced in each bone model (n = 54) from CORA zero in the mechanical axis. The rotation point for creating the deformity was located at the cortical bone level on the concave side of the deformity. Group I (n = 18), the study group, represents the calculations made using the simplified method proposed in the study, called circumferential method (CC). Group II (n = 18), the control group, made of the calculations performed by AutoCAD^tm^, called, in this study, the exact calculation (EC). Group III (n = 18), also considered as control group, was made up of the calculations performed by the classic trigonometric method (TM).

The length of the wedge base calculated on the exact group through AutoCAD^tm^ software, considered an accurate method of correcting the mechanical axis of the bone model, was used as a reference for comparing the length of the bone wedge base with CC and TM groups.

### Description of calculations

**Figure 1:**
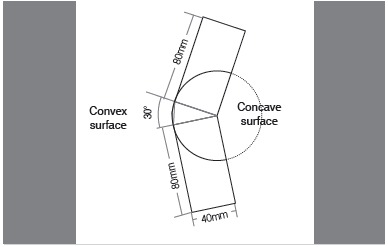
Model used as standard. In this example, the hypothetical angle of the bone deformity was 30°.

**Figure 2:**
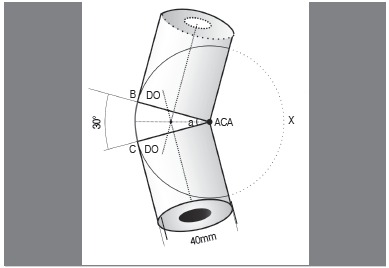
Model used for defining the circumferential calculation with a hypothetical angle of 30°, with rotation plane on the concave surface of the deformity.

A) Circumferential Calculation (CC): Initially, we used as reference measurements for all calculations two rectangles representing the flat projections of the proximal and distal portions of any long bone, producing 18 angular deformities (sample size), ranging from 5° to 90° with 5° intervals between them. The rotation point was positioned in one of the ends of the model configuring, at the end, a convex surface and a concave surface. For these models we set a standard bone diameter of 40mm. (Figures 1 and 2)

Using the standard model described above, the circumferential calculations were performed in order to find the base of the bone wedge needed for correction of the angular deformities. This wedge, with an angle (a) can be defined through the arc measures of a circle (x), whose center is on the concave surface of the deformities (closing wedge) and whose radius corresponds exactly to the bone diameter (DO). Thus, as the arc of any circle is equal to 2.π.r, in radians, the arc of the circle (BC) corresponds, in degrees, to:

- BC = 2 x π x Radius x wedge angle/360°
- BC = 2 x π x DO x a/360° (We have defined the constant values 2π/360 as "E")
- Therefore, BC = E x DO x a, where constant E = 0.0174

B) Exact Calculation (EC): The exact calculation of correction of hypothetical bone deformities were held using the software AutoCAD^tm^ using the same sample size of 18 models of hypothetical bone deformities, the same values ​​of angular deformities (5-90°) and the same hypothetical bone diameter (DO) of 40mm used in the circumferential calculation (CC).

**Figure 3:**
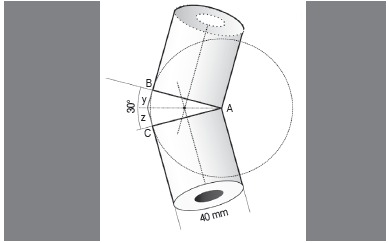
Model used for defining the exact dimensions of the wedge through AutoCADtm software in a deformity with a hypothetical angle of 30°.

In this group, the base of the osteotomy corresponds to two projections in the Cartesian axis y and z. (Figure 3) Therefore, it has been defined that the wedge base (BC) used in this study corresponded to the sum of the lateral projections (Y and Z) of the convex side of the deformity, according to the schematic drawing. (Figure 3)

**Figure 4:**
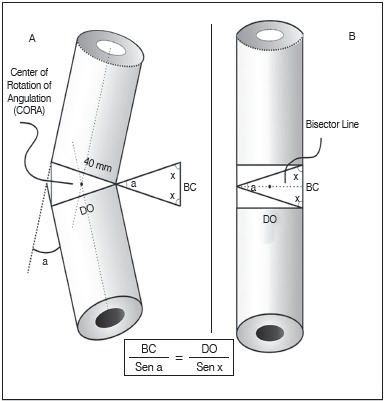
(A) Model used to calculate the base length of an opening cuneiform osteotomy, with angle of 30° and bone diameter 40mm. (B) Same model after the opening cuneiform osteotomy and deformity correction. Note the trigonometric formula used to calculate the wedge base (BC).

C) Trigonometric calculation (TC): The calculation of the base of the bone wedges in the 18 hypothetical models by TC was performed by applying the law of sines in any triangle.^11^ ^,^ ^17^ Thus, we have considered a bone deformity corresponding to the angle (a), whose wedge base is formed on the concave side of the deformity after simple osteotomy through the bisecting line through the deformity apex (CORA) and the rotation axis (ACA) over the convex side of the bone, producing an opening wedge, thus, BC can be calculated by the following formula:

x = (180 - a)/2, therefore, BC = DO x Sen (a)/Sen {[180 - a]/2}. (Figure 4A and 4B)

The calculation of the length of the bone wedge base, aiming the correction of deformities were performed on 18 bone models of each group, using the specific method of calculation of each group. 

### Statistical analysis 

The collected data were originally stored in Excel charts, version 10.0 (Microsoft^tm)^ and AutoCAD^tm^.

Quantitative variables (length of the wedges calculated by the exact method (AutoCADt^m)^, the trigonometric method [law of sines] and circumferential method) were expressed by their average values, standard deviation and minimum and maximum values. We used Tukey's statistical test (parametric test), after confirmation that the samples were normally distributed. The significance level alpha was equal to 5% (or *p* <0.05).

Static analysis of the data was performed using the software Prisma^tm^, version 5.0 for Windows^tm^.

## RESULTS

In the 18 hypothetical bony angular deformities, the average lengths of corrective wedges obtained by trigonometric, circumferential and exact method were respectively 32.21 ± 16.81mm, 33.16 ± 18.63mm and 35.22 ± 23.52mm. There was no statistically significant difference between these three calculation methods (*p*>0.05).

Table 1 shows the distribution of values related to the measures of the wedge bases using the exact method by AutoCAD^tm^, the circumferential method proposed in this study and the trigonometric method using the law of sines.

**Table 1:** Results of measurements (mm) of the length of corrective bone wedges obtained by exact calculation (AutoCADtm), circumferential and trigonometric methods.

Deformity angle (degrees)	Measurements (mm)		
	Exact method (AutoCAD)	Circumferential method	Trigonometric method
5	3.493	3.491	3.490
10	6.99	6.98	6.97
15	10.53	10.47	10.44
20	14.10	13.96	13.89
25	17.73	17.45	17.31
30	21.43	20.94	20.70
35	25.22	24.43	24.05
40	29.11	27.92	27.36
45	33.13	31.41	30.61
50	37.30	34.90	33.80
55	41.64	38.39	36.94
60	46.18	41.88	40.00
65	50.96	45.37	42.98
70	56.01	48.86	45.88
75	61.38	52.36	48.70
80	67.12	55.85	51.42
85	73.30	59.34	54.04
90	80.00	62.83	56.56
Mean	35.22	33.16	32.21
SD	23.52	18.63	16.81
***p***		0.6364	

SD: Standard deviation; p Tukey's test; p>0.05, there was no statistically significant difference.

**Figure 5:**
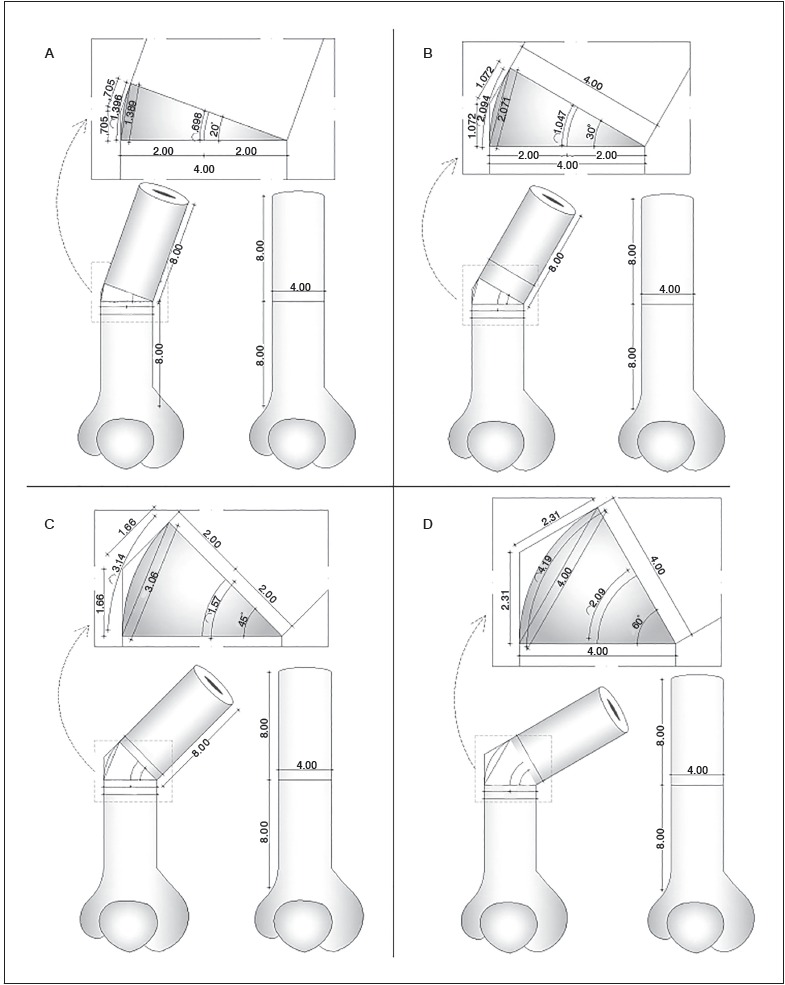
(A, B, C and D). Examples of four hypothetic models produced in AutoCADtm of femurs with angular deformities corrected through bone wedges which lengths were calculated by the three methods presented in this study. (A) Bone deformity of 10°; (B) Bone deformity of 30°; (C) Bone deformity of 45°; (D) Bone deformity of 60°.

Figure 5 (A, B, C and D) represents an example of four bone models created in high precision by AutoCAD^tm^, containing the results of calculations for corrective wedges in a hypothetical femur according to the three methods presented in this study.

## DISCUSSION

The surgical planning is an essential part to obtain satisfactory results in cuneiform osteotomies; therefore, obtaining accurate measurements preoperatively are even more indispensable.^8^ ^-^ ^10^ Thus, although it was not the object of this study, the planning of deviations corrections of the mechanical and anatomical axis of the upper or lower limbs must include a thorough radiographic study in at least two orthogonal views, which must be carried out carefully so that the practical results are as reliable as possível.^8^ ^-^ ^10^ ^,^ ^18^ ^,^ ^19^

Thus, it is essential to distinguish two required parameters through these radiographic images, not only for proper correction of a bone deformity, but also to understand how the authors concluded the formula of the CC method proposed in the study. These are the rotation angle center named CORA (Center of Rotation of Angulation) with its corresponding transverse and longitudinal bisector lines and the axis of angulation correction, ACA (Angulation Correction Axis).^8^ ^-^ ^10^ CORA is defined as the point of intersection of lines parallel to the longitudinal axis of the proximal and distal diaphyseal axis of the bone under study and the lines that divide in half the angle formed by these projections corresponding to the transversal and longitudinal bisectors.^8^ ^-^ ^10^ ACA is represented by a point on the flat projection of a deformity and to avoid the correction to cause translations as the final result of a cuneiform osteotomy, it should be on any point contained on the line representing the transversal bisector of CORA.^8^ ^-^ ^10^ ^,^ ^16^ ^,^ ^19^

Besides the interpretation of CORA and ACA, in order to reach the calculation method proposed by this study, we used circumferential planar geometry concepts to define the corrective wedges bases without using sine, cosine, and tangent tables.^20^ Therefore, we took into consideration that the movement to correct angular deformities, in principle, follows the circular momentum rules, since the proximal and distal bone segments are referentially moved around ACA. ACA, which is represented by a fixed point in plane geometry, produces geometric figures whose virtual dimensions are similar to the elements of a circumference.^20^ The angular circumference representations, in turn, consist of "arches" of measures easily calculable and potentially useful.^6^ ^,^ ^20^ As expected, these concepts showed to be applicable and the circumferential calculation formula has also proven to satisfactorily define the dimensions of the osteotomy wedges, with attested good results, besides being more easily performed than trigonometric calculations.

Using as deformity standard progressive angles produced between two planar cylinders, it has been found that as the deformities increased, the distortions between TC and CC calculation methods to define the base of wedge osteotomies were also bigger. Despite the results presented in this study indicate the absence of significant differences between these measurements, regarding its practical use, it is known that differences larger than 5mm between methods, such as those encountered in deformities above 65°, could generate excessive or incomplete correction, depending on their application. However, although the exact calculation used as standard in this study takes into consideration that convex side deformities would resemble the junction of the lines projected by the proximal and distal cortical (y and z in Figure 3), in practice, whether congenital or acquired, bone deformities usually have rather "rounded" convex surfaces which would make their flat projections also similar to an arc of circumference. This fact leads to repercussions especially in cases where one chooses to correct a deformity through subtraction wedges. The calculated bone wedge base is initially applied to delimit the sectional area of ​​the convex apex of the curve, through a flexible millimetric reference. That, in turn, should be properly shaped to the bone surface prior to marking these limits and cutting. The importance of obtaining measures similar to the arc of a circle to ensure the accuracy of results stands out; the circumferential calculation has been presented as an excellent option regarding resection wedges.

For deformities requiring correction by opening osteotomy, the wedge graft base formed after bone opening may be measured between the two cortical ends as a straight line, although the movement is around a fixed axis (ACA). Thus, CT and CC are excellent choices, especially if the deformity on the convex bone surface resembles a line in the planned study.

Me may state that using a simple method to define the dimensions of corrective wedges can facilitate the performance of osteotomies and positively influence its practical results, although there are other calculating methods and the wide availability of computer graphics for surgical planning nowadays. The possibility of avoiding trigonometric tables is still a facilitator that showed not to influence the outcome of corrections of uniplanar deformities through cuneiform osteotomy. 

## CONCLUSION

The method used to calculate the circumferential length of the base of the bone wedge aiming correction of uniplanar angular deformity of long bones is an effective and user-friendly method, as compared to the accurate and trigonometric methods. It can be easily reproduced in humans with great safety in the presurgical planning for correcting uniplanar angular deformities of long bones. However, studies with experimental synthetic bone models or cadavers are needed for later use in humans.
